# The route to employability: a longitudinal study on a sample of Italian job seekers

**DOI:** 10.1007/s10775-021-09482-3

**Published:** 2021-06-05

**Authors:** Alessandro Lo Presti, Assunta De Rosa, Monica Zaharie

**Affiliations:** 1 Dipartimento Di Psicologia, Università Degli Studi Della Campania “Luigi Vanvitelli”, Viale Ellittico, 31, 81100 Caserta, Italy; 2 Faculty of Economics and Business Administration, Babeș-Bolyai University, Cluj Napoca, Romania

**Keywords:** Employability, Proactive personality, Core self-evaluations

## Abstract

This study aimed to examine the main predictors of employability, building on a recent conceptual model on employability developed by Lo Presti and Pluviano (Organ Psychol Rev 6(2): 192–211, 2016). Survey based data were collected from a sample of 263 Italian job-seekers through a longitudinal study. The results revealed that employability was more strongly determined by personal dispositions than by external factors, such as life circumstances and that the variables with the most impact were proactive personality, core self-evaluations, and educational level, rather than employability culture, family employability support, and previous work experience. The paper reveals an understanding of the relative importance of antecedents that determine employability.

## Résumé

**La route vers l'employabilité : Une étude longitudinale sur un échantillon de demandeurs d'emploi italiens**

Cette étude visait à examiner les principaux prédicteurs de l'employabilité, en s'appuyant sur un modèle conceptuel récent de l'employabilité développé par Lo Presti et Pluviano (Organ Psychol Rev 6(2): 192–211, 2016). Des données basées sur des enquêtes ont été recueillies auprès d'un échantillon de 263 demandeurs d'emploi italiens dans le cadre d'une étude longitudinale. Les résultats ont révélé que l'employabilité était plus fortement déterminée par les dispositions personnelles que par des facteurs externes, tels que les circonstances de la vie, et que les variables ayant le plus d'impact étaient la personnalité proactive, les auto-évaluations fondamentales/de base et le niveau d'éducation, plutôt que la culture de l'employabilité, le soutien familial à l'employabilité et l'expérience professionnelle antérieure. Le document révèle une compréhension de l'importance relative des antécédents qui déterminent l'employabilité.

## Abstract (de)

**Der Weg zur Beschäftigungsfähigkeit: Eine Längsschnittstudie an einer Stichprobe von italienischen Arbeitssuchenden**

Ziel dieser Studie war es, die wichtigsten Prädiktoren der Beschäftigungsfähigkeit zu untersuchen, aufbauend auf einem kürzlich von Lo Presti und Pluviano (Organ Psychol Rev 6(2): 192–211, 2016) entwickelten konzeptionellen Modell zur Beschäftigungsfähigkeit. Es wurden umfragebasierte Daten von einer Stichprobe von 263 italienischen Arbeitssuchenden im Rahmen einer Längsschnittstudie erhoben. Die Ergebnisse zeigten, dass die Beschäftigungsfähigkeit stärker durch persönliche Dispositionen als durch externe Faktoren, wie z. B. Lebensumstände, bestimmt wurde und die Variablen mit dem größten Einfluss waren die proaktive Persönlichkeit, zentrale Selbsteinschätzungen und das Bildungsniveau, und nicht die Kultur der Beschäftigungsfähigkeit, die familiäre Unterstützung der Beschäftigungsfähigkeit und frühere Arbeitserfahrung. Die Arbeit zeigt ein Verständnis der relativen Bedeutung von Antezedenzien, die die Beschäftigungsfähigkeit bestimmen.

## Resumen

**El camino a la empleabilidad: Un estudio longitudinal con una muestra de desempleados italianos**

Este estudio pretende examinar los principales predictores de empleabilidad, construyendo un modelo conceptual de empleabilidad desarrollado por Lo Presti y Pluviano (Organ Psychol Rev 6(2): 192–211, 2016). La información fue recogida a través de una encuesta realizada a 263 Italianos en situación de búsqueda de empleo, a través de un estudio longitudinal. Los resultados revelaron que la empleabilidad estaba más fuertemente determinada por la disponibilidad personal que por factores externos como las circunstancias vitales. Las variables con más impacto fueron la personalidad proactiva, las autoevaluaciones básicas y el nivel educativo, y no tanto la cultura de ocupabilidad, el soporte familiar a la empleabilidad y las experiencias de trabajo previas. El artículo revela la importancia relativa de los antecedentes que determinan la empleabilidad.

## Introduction

Unemployment and re-employment have always occupied a significant place in political, economic, and scholarly debate (Forrier et al., 2018). Recurrent economic crises have reduced access to the labour market for wider sections of the active population and have led to reduced wages and incomes and a subsequent vicious circle made of a drop in consumption, tax payments, and state investments (McQuaid & Lindsay, 2005). In the last quarter of 2019, the unemployment rate in EU27 was 6.3%, with an a posteriori estimate of a rapid and dramatic deterioration caused by the Covid-19 pandemic’s economic effects. The three countries with the highest levels of unemployment in the EU27 are Greece (16.7%); Spain (14.2%), and Italy (9.7%) (Eurostat 2019). The effects of unemployment on individuals have been widely studied, as it has been associated with negative health consequences (Norström et al., 2019), increased anxiety, depression, and adverse effects on self-perception and physical health (McKee-Ryan et al., 2005). Thus studies with a preventive focus are of great importance.

In these circumstances, many have seen *employability* as an answer to the need to foster the individual occupational chances in an increasingly turbulent labour market, preventing the risk of prolonged unemployment (McArdle et al., 2007). Based on the premise that employability can be considered a resource, Lo Presti and Pluviano (2016) have recently advanced a conceptual model that conceives employability as an individual resource for career success, aiming to provide a complete framework for the study of antecedents and consequences of employability. Until now, there has been little empirical research addressing this model, and further evidence is being called for (Lo Presti et al., 2019). Moreover, despite being acknowledged as critical for individuals seeking employment (Berntson et al., 2006), employability has been mainly investigated in the context of employees (Kirves et al., 2013; Van der Heijden et al., 2009) and students (Cheung et al., 2018; Gunawan et al., 2020; Herbert et al., 2020). Employability is rarely researched in relation to more disadvantaged groups, such as the unemployed (Arendt et al., 2020; Cheng et al., 2020). The few available studies have mainly examined its outcomes: job search behaviours (Cheng et al., 2020; Fernández-Valera et al., 2020; McArdle et al., 2007), well-being (Vanhercke et al., 2015), and re-employment chances (Koen et al., 2013), whilst less attention has been paid to the examination of its antecedents: proactive personality, boundaryless mindset, career self-efficacy, identity awareness, social support (McArdle et al., 2007), and emotional competencies (Hodzic et al., 2015).

Based on the conceptual model developed by Lo Presti and Pluviano (2016), the current study aims to examine the predictive role of six potential antecedents of employability in a sample of Italian individuals looking for a job, through a longitudinal research design, examining antecedents at time 1 (t1), and employability four months later (t2). The contributions of this study are threefold. First, our study responds to the Lo Presti and Pluviano’s (2016) call for testing their theoretical propositions, providing an alternative and more integrative framework to employability. Second, to the best of our knowledge, few studies have examined employability in relation to unemployed people, who represent an under-researched group in the frame of employability and a significant research gap to be filled considering the potential positive effects associated with increased employability. Third, despite previous scholarly efforts, the study of employability predictors still appears limited (Wittekind et al., 2010) and not systematized. Moreover, the recent work by Gunawan et al. (2020) argues the relevance of personal and situational factors as predictors of employability and calls on the need for further research to explore these predictors. Therefore, it is our aim to jointly investigate the association of a wide array of antecedent variables with employability, providing evidence on the predictive role of different factors on employability.

The evidenced predictive roles of the antecedents on employability may also have significant practical implications for different stakeholders: employers, job-seekers, and their families. In particular, interventions at the organizational (e.g., training courses), family (e.g., family counselling), and individual (e.g., career counselling or guidance) levels may be implemented in order to promote these factors amongst job-seekers through the engagement of different agents: outplacement services, job centres, career counsellors or relatives.

### Conceptualizing employability

Early studies approached employability as the ability to obtain and maintain a job without recognizing the several and dynamic dimensions that characterize it in the current context (Gazier, 1998). More recently, we have witnessed dramatic changes brought about by globalization and digitalization. Organizations have been forced to adapt, increasing the possibility that individuals have to experience multiple and more frequent occupational transitions (Guilbert et al., 2016). Thus, employability is no longer considered important only for supporting individuals looking for a job, but as a personal resource that can help individuals being more responsible in directing their careers and promoting their overall quality of life (Van der Heijde & Van der Heijden, 2006). In fact, as the traditional organizational career model is less accessible and feasible for more and more workers (Clarke, 2013), new career conceptualizations (DeFilippi & Arthur, 1994; Hall, 1996) and new models and definitions of employability (Forrier & Sels, 2003a; Van der Heijde & Van der Heijden, 2006) have been proposed, focusing on the individual's ability to be responsible for one’s career, to be highly adaptable, and to search for personal fulfilment and career satisfaction. Employability has been more frequently associated with job-search intensity (Cheng et al., 2020), global health and mental well-being (Berntson & Marklund, 2007), subjective and objective career success (Van der Heijden et al., 2009), and job satisfaction (Gowan, 2012), and is predicted, amongst other things, by perceived mobility and optimism (Kirves et al., 2013), volition and self-efficacy (Ngo et al., 2017).

More recently, Lo Presti and Pluviano (2016: 196) claimed that employability is “a personal resource that individuals develop across their working lives aimed at increasing one’s career success, both attaching importance and committing to making sense of past work experience and envisioning one’s professional future, acquiring valuable competencies and skills, improving their formal and informal career-related networks, exploring their social environment in search of opportunities and constraints to their career pathway”. Considering employability as a resource, Lo Presti and Pluviano (2016) made explicit reference to the Conservation of Resources theory (Hobfoll, 1989), whose main tenet is that people strive to protect and build resources and avoid the potential loss of these resources. Highly enterprising individuals are thought to be healthier than those with fewer resources, adapt more proactively to their jobs, and achieve their goals successfully (Hobfoll, 1989).

Lo Presti and Pluviano (2016) advanced a configurational model of employability, defining its components and proposed a causal model identifying career success as a proximal outcome and three clusters of potential antecedents, namely: training and work experiences, life events and circumstances, and dispositions. *Training and work experiences* refer to those experiences that foster the development of general human capital (e.g., educational attainment and cognitive skills) and job-specific human capital (i.e., attributes that foster performance in specific jobs). In the current study, we refer to the educational level and the number of previous job roles as proxies of training and work experiences. *Life events and circumstances* are those external conditions that cannot be directly controlled by the individual but can influence employability. Lo Presti and Pluviano (2016) stated that they include personal (e.g., chronic illness), household (e.g., parenthood, family support), organizational (e.g., underemployment, mentorship), and social (e.g., economic depression, welfare policies) events and circumstances. In the present study, we examined family employability support and employability culture as proxies of life events and circumstances. Finally, *personal dispositions* pertain to those personality characteristics that may foster or hinder the development of employability. In this study, we examined core self-evaluations and proactive personality as proxies of personal dispositions.

Up to now, limited empirical evidence is available about this theoretical model. Recently, the configurational model of employability has found empirical support as a newer scale has been developed and validated (Lo Presti et al., 2019), providing evidence about its construct validity and its predictive validity with regard to career success. However, the causal model has not been empirically tested yet. This study provides a first empirical attempt as it focuses only on employability’s antecedents.

### Antecedents of employability

As for *training and work experiences* predictors, human capital and its investment (i.e., education and training experiences) have always occupied a central role in the literature (Bertson et al., 2006; Wittekind et al., 2010). Based on Becker's studies (1993), Williams and Krasniqi (2018) outlined human capital as the individuals’ skills and knowledge fuelled by education and training. Early literature argued that workers with a higher educational level have higher occupational chances (Mincer, 1991). However, despite the urge to track the education impact on individuals’ outcomes on the labour market (Pavlin & Svetlik, 2014), there is sparse research on the relationship between education and employability, mainly limited to samples of employees. Furthermore, the existing findings on education and employability are also contradictory. Wittekind et al. (2010) found that education measured as college vs. no college degree negatively predicted employees’ employability, whilst Nauta et al. (2009) showed no relationship between education and employability. On the other hand, Berntson et al. (2006) and Juhdi et al. (2010) found education to be positively associated with perceived employability. Focusing on education during unemployment, Muehlboeck et al. (2020) showed that only the long-term programs positively impact employability beyond the duration of the activity. Whilst in some studies, human capital has been examined as an employability dimension (Fugate et al., 2004), in this study, educational level is considered as a predictor of employability. The importance of individual experiences at work is central in both research and practice, being extensively studied amongst graduates (Helyer & Lee, 2014) and sparsely amongst employees (Judhi et al., 2010). Work experience has been studied as time spent on the job or tenure (McDaniel et al., 1988) or as the number of times that a particular task has been performed (Vance et al., 1989). Judhi et al. (2010) focused on the factors that could affect employability, analyzing the association between tenure and external-internal employability and suggesting that a greater work experience increases the individual's chances in the labour market. Also focused on employees, León and Morales (2019) revealed differences in the impact on employability as a function of the tenure characteristics, whilst Irwin et al. (2019) showed no impact of the duration of the students’ work experience. Whilst the lack of work experience has been traditionally indicated as a challenge when entering employment for young individuals, little is known about the impact of the number of job experiences on job seekers’ employment.

The cluster *life events and circumstances* includes those external conditions that can influence employability and that are beyond the individual control (Lo Presti & Pluviano, 2016). They can include organizational (e.g., significant mentorship experiences), family (e.g., family conditions preventing from adequate schooling), social experiences (e.g., being raised in a poor neighbourhood), that had a significant impact on the individual and thus on his/her subsequent employability. Berntson et al. (2006, p. 226) stressed the role of the dual labour market paradigm, which suggests “that labour market opportunities and restrictions are crucial in determining an individual’s employability” and the role of the economic situation in employability. The economic situation is significantly impacting employability as individuals exposed to a more stimulating work environment reported higher employability, as well as individuals living and working in metropolitan areas. Other literature models highlight the importance of situational factors (Hogan et al., 2013; McQuaid & Lindsay, 2005) as antecedents for perceived employability (Cheung et al., 2018). Employability culture can be defined as the support offered by organizations to stimulate the orientation towards employees’ employability, by developing flexible skills, the ability to adapt to changes, and the willingness to perform different tasks and roles (Nauta et al., 2009). Based on this definition, Nauta et al. (2009) argued that human resource managers need to engage in interventions that support the organization to achieve its targets and, at the same time, develop the employees’ flexibility. Several studies suggested that organizational support has a positive impact on employability (De Vos et al., 2011; Nauta et al., 2009) consistently with the proximal environmental resources category of career success predictors proposed by Spurk et al. (2019). Whilst social support from significant others such as teachers and peers was proved to impact perceived employability (Cheung et al., 2018), the family support for employability remains an underexplored area of ​​study. In this paper, we examined family support for employability operationalised as the individuals’ perception of the support provided by the family as a resource to deal with the challenges faced in their professional life. However, whilst several studies have examined the impact of the family on work, there is a void in the literature that explores the relationship between family support and employability. Some studies suggested that family support may influence employment choices and career development (Beauregard, 2007; Lindstrom et al., 2007). Other studies showed that family support affects both career self-efficacy beliefs and career decision-making (Ferry et al., 2000). The family seems to play a central role in the development of self-efficacy by encouraging active exploration of the environment, which supports individuals in developing stable identity structures that will allow them to better cope with the environment (Ryan et al., 1996). The literature distinguishes structural and process family variables that can influence an individual's career development. The former refers to demographic variables, including parents’ education, employment, and socio-economic status, whilst the process variables refer to values, expectations, and support (Lindstrom et al., 2007). The family structural variables “seem to be an especially strong predictor of later access to career opportunities and options” (Lindstrom et al., 2007, p. 349). Parents with higher scores in structural variables are more likely to provide tools and emotional support to the individual (Blustein, 2002). However, it appears that family process variables have a greater influence on career development than family structural variables (Whiston & Keller, 2004).

More considerable attention has been given to the study of *dispositions* in the workplace. Scholars emphasized that “individuals need a certain set of skills, competencies, and personality attributes to make them more employable” (Potgieter et al., 2012, p. 583). Potgieter et al. (2012) showed that personality attributes were significantly and positively related to general employability attributes. This study focuses on core self-evaluations and proactive personality. We define core self-evaluations as "basic assessments that people make of themselves" (Judge, 2009: 58). Core self-evaluations are a set of personality aspects such as self-esteem, self-efficacy, locus of control, and lack of neuroticism (Judge et al., 2003). The individuals’ positive self-assessment allows them to have greater control of the situation, to be more effective in a variety of situations, to proactively use coping strategies in stressful situations by predicting positive results on work performance and life satisfaction (Judge et al., 2004). In general, individuals with positive core self-evaluations have the ability to control their environment; on the contrary, those with negative core self-evaluations lack confidence in themselves and in their skills (Judge & Bono, 2001). Available evidence shows the positive effects of core self-evaluations on life satisfaction (Piccolo et al., 2005) and mental-physical health and turnover intentions (Virga et al., 2017). Özer et al. (2016) found that core self-evaluations predicted life satisfaction. Stressing the lack of findings on how dispositional factors and, in particular, core self-evaluations impact employability, Onyishi et al. (2015) found a significant association between students’ core self-evaluations, preparation of job search behaviour, and perceived employability. Proactive personality refers to the individuals' disposition to actively modify the environment to achieve their goals (Crant, 2000). Proactive individuals are able to identify opportunities and act on them, as opposed to passive personalities that adapt to circumstances without changing them (Seibert et al., 1999). The proactive personality has been extensively studied. Crant (1995) found that proactive personality was predictive of objective job performance; in a further study, Seibert et al. (1999) found a positive association between proactive personality and objective measures of career success (i.e., current salary and promotions). Brown et al. (2006) examined the association between proactive personality and job search behaviour in a longitudinal study with a sample of graduating college students, showing that proactive personality represents a significant antecedent of job search. Except for the findings on graduating students (Gunawan et al., 2020), proactivity has been examined less frequently in employability studies. Van Dam (2004) argued that personality traits are important antecedents of employability and found that openness and initiative related positively to employability orientation, suggesting that having an open mind to change and being proactive is essential for maintaining one's internal employability. Assuming that a proactive personality is helpful to have control and respond effectively to the environment, we propose that it may foster individual employability. Through active behaviours, proactive individuals would be able to grasp information and opportunities in the environment and better act towards achieving their goals.

### Study aim and hypotheses

Consistently with Lo Presti and Pluviano (2016), the aim of the present study is to examine the association of a wide array of antecedents variables with employability. Based on the abovementioned empirical evidence as well as theoretical considerations, we advance a series of hypotheses. Firstly, we assume that educational level will be positively associated with employability (H1), based on the claim that training and work experience may promote the acquisition of skills and abilities useful to the individual’s employability (Berntson et al., 2006). Second, we assume that the number of previous job roles will be positively associated with employability (H2), as we expect that individuals with more work experiences would have developed skills and knowledge that can be valuable in the labour market and therefore have higher chances of being employable (Judhi et al., 2010). Following, we predict that employability culture will be positively associated with employability (H3), as it can be argued that organizations that promote an employability culture stimulate the employability of their employees, encouraging them to develop their skills (Nauta et al., 2009). Moreover according to Whiston and Keller (2004) we hypothesize that family employability support will be positively associated with employability (H4), as it can be argued that family influences the individual's ability to engage in pursuing career opportunities and options as it provides both emotional and instrumental support, thus increasing their employability. Later, we mentioned that scholars have given wide attention to the study of dispositions (Potgieter et al., 2012) as antecedents of employability, so we predict that core self-evaluations will be positively associated with employability (H5), because, considering that core self-evaluations define an individual ability to control the environment and being more effective, it can be argued that it makes the individual more able to gather information from the environment necessary to actively respond of workplace’s needs, thus being fostering one’s own employability (Onyishi, et al., 2015). Finally, we hypothesize that Proactive personality will be positively associated with employability (H6), based on the claim that managing one’s own career is became a central phenomenon in the current workplace (Gunawan et al., 2020), so that it can be argued that a proactive personality is useful for identifying opportunities and adapting in different situations to achieving career goals.

## Method

### Participants

762 unemployed Italian job-seekers, voluntarily recruited via a convenience sampling strategy within job centres, were initially recruited (Time 1). After careful inspection of these returned questionnaires, 22 cases were removed because they were filled out by employed individuals. At Time 2, 263 questionnaires were returned (response rate = 35%). As subsequent analyses are carried out on the respondents that filled both t1 and t2 questionnaires, the following information refers to *N* = 263, out of which 143 (54.4%) were men and 120 (45.6%) were women, with an average age of 34.22 years (*SD* = 11.65). The average educational level was 15.04 years (*SD* = 3.97), which means that, on average, most of them had a high school diploma and attended the university to some degree. The average general tenure was 9.60 years (*SD* = 10.36), whilst participants had, on average, 2.73 previous job roles (*SD* = 2.28). Participants who completed only the Time 1 questionnaire were compared to those who completed both questionnaires, and no statistically significant differences emerged in regards to socio-demographic variables.

### Measures

*Educational level* was measured as the total number of years spent in education.

*Work experience* was measured as the participants’ number of previous job roles.

#### Employability culture

The scale by Nauta et al. (2009; Italian version by Lo Presti & Elia, 2020) was used. It consisted of eight items (e.g., “The organizations I worked for encouraged employees to broaden their skills”) that were adapted to refer to previous significant organizational experiences (and not only to the current organization, as the original scale) and were assessed through a 5-point Likert scale (1 = “strongly disagree”, 5 = “strongly agree”). Cronbach’s alpha was .80.

#### Family employability support

Three items (e.g., “My family has always supported me in my education and training”, “My family has done its best to allow me to better face the world of work”, “I have always found advice and support in my family in regards to my professional life”) were developed for this study. Responses were assessed through a 5-point Likert scale (1 = “strongly disagree”, 5 = “strongly agree”). Cronbach’s alpha was .93.

#### Core self-evaluations

The scale by Judge et al. (2003; Italian version by Di Fabio & Busoni, 2009) was used. It consists of twelve items (e.g., “I am capable of coping with most of my problems”) that were assessed through a 5-point Likert scale (1 = “strongly disagree”, 5 = “strongly agree”). Cronbach’s alpha was .84.

#### Proactive personality

The scale developed by Seibert et al. (1999; Italian version by Trifiletti et al., 2009) was used. It consists of ten items (e.g., “Wherever I have been, I have been a powerful force for constructive change”) assessed through a 5-point Likert scale (1 = “strongly disagree”, 5 = “strongly agree”). Cronbach’s alpha was .87.

#### Employability

We used the scale developed by Lo Presti et al. (2019). The scale consists of 28 items (e.g., “Developing new competencies about my occupation is easy to me”) that were assessed through a 5-point scale (0 = “not at all”, 4 = “completely”). Cronbach’s alpha was .94.

### Procedure

This study used a self-report questionnaire that was delivered and collected by trained researchers. Job-seekers participants were recruited at the job centres they visited for different needs as: looking for job-offers, having job-interviews, resolve bureaucratic issues (e.g., unemployment benefit), and were asked to complete the Time 1 questionnaire during waiting times. The first page of this questionnaire contained the aims of the study, the instructions for participation, and the scales assessing study predictors (educational level, no. of job roles, employability culture, family employability support, core self-evaluations, proactive personality) as well as control variables. After about 4 months, participants received by e-mail a second questionnaire assessing employability (Time 2).

As for ethical issues, this study adheres to the Helsinki Declaration (World Medical Association, 2001). Moreover, all study participants provided their informed consent consistently with the Italian laws of data protection (legislative decree n.196/2003).

### Analyses

First, missing values (0.004%) for continuous variables were replaced through their Expected Maximization method (Schlomer et al., 2010). Cronbach’s alphas were used to assess the scales’ internal consistency, whilst means and standard deviations were used as descriptive statistics. Associations between variables were described recurring to point-biserial (for gender) and zero-order correlations (for continuous variables), and hierarchical linear regressions. Dominance analysis was computed to rank order by importance of the predictors of employability. Dominance analysis relies on estimating an *R*^2^ value for all possible combinations of predictors as they relate to a dependent variable (Azen & Budescu, 2003). Dominance analysis is needed for determining if a predictor is “dominant” over another predictor: that is, a predictor’s additional contribution in terms of explained variance is greater than the contribution of the competitor predictor (Darlington & Hayes, 2016).

## Results

Table 1 depicts descriptive statistics and zero-order correlations amongst study variables. In particular, employability positively correlated with family employability support (*r* = .15, *p* = .01), core self-evaluations (*r* = .36, *p* < .001), and proactive personality (*r* = .37, *p* < .001).

**Table 1:** Descriptive statistics and zero-order correlations

	*M* (*SD*)	1	2	3	4	5	6	7	8
1) Gender^1^	–								
2) Age	34.22 (11.65)	− .10							
3) Educational level	15.04 (3.97)	− .01	− .38***						
4) No. of job roles	2.73 (2.28)	− .04	.31***	− .11					
5) Family employability support	3.75 (1.15)	− .04	− .04	− .03	− .08				
6) Employability culture	3.09 (.75)	− .01	.06	− .08	− .05	.32***			
7) Core self-evaluations	3.34 (.61)	− .07	.22***	− .14*	.10	.36***	.30***		
8) Proactive personality	3.69 (.62)	.01	.15*	− .12*	.02	.31***	.18**	.50***	
9) Employability	2.65 (.61)	− .02	.11	.04	− .04	.15*	.02	.36***	.37***

^1^1 = male, 2 = female; * *p* < .05; ** *p* < .01; *** *p* < .001

Employability was regressed on study variables (Table 2), controlling for gender (β =  < .01, *ns*), and age (β = .10, *ns*). Employability was positively predicted by educational level (β = .13, *p* = .035), core self-evaluations (β = .26, *p* < .001), and proactive personality (β = .26, *p* < .001), whilst the number of job roles (β = -.09, *ns*), employability culture (β = -.12, *ns*), and family employability support (β = .02, *ns*) were not significant predictors. Predictors explained 21% of employability variance.

**Table 2:** Employability regressed on study variables

	Employability			
	Step 1	Step 2	Step 3	Step 4
Gender^1^	− .01	− .01	< .01	< .01
Age	.11	.17*	.18**	.10
Educational level		.10	.10	.13*
No. of job roles		− .08	− .07	− .09
Family employability support			.17**	.02
Employability culture			− .04	− .12
Core self-evaluations				.26***
Proactive personality				.26***
*F*	1.68	1.78	2.41*	8.61***
*R*^2^	.01	.03	.05	.21
Δ*R*^2^		.01	.03*	.16***

^1^1 = male, 2 = female

*p < .05; **p < .01; ***p < .001

Dominance analysis (Table 3) was carried out to identify the differential predictive role of several variables on employability. The table depicting all possible R^2^ combinations of predictors over employability is available upon request from the first author.

**Table 3:** Dominance matrix of employability’s predictors

	*R*^2^	1	2	3	4	5	6
(1) Educational level	.001	–	.94	.75	.63	.00	.00
(2) No. of job roles	.002	.06	–	.63	.12	.00	.00
(3) Family employability support	.02	.25	.37	–	.25	.00	.00
(4) Employability culture	.0003	.37	.88	.75	–	.00	.00
(5) Core self-evaluations	.13	1	1	1	1	–	.06
(6) Proactive personality	.14	1	1	1	1	.94	–

Furthermore, the predictors accounted for approximately 20.6% of employability variance. In absolute terms and ascending order, employability culture, educational level, and the number of job roles played an insignificant role in explaining employability variance (about 0% of the employability variance), family employability support explained about 2.4%, core self-evaluations about 13%, and proactive personality about 14.1% of the employability variance.

Table 3 depicts the dominance matrix, which shows the proportion of regression sub-models in which the inclusion of the predictor in the row results in a larger increase in R^2^ than the inclusion of the predictor in the column. For instance, for the first row, education level is dominant over the number of job roles (0.94), family employability support (0.75), employability culture (0.63), whilst it is not dominant over core self-evaluations (< 0.01) and proactive personality (< 0.01). Amongst the most dominant variables, proactive personality is dominant over all variables, whilst core self-evaluations over all variables except for proactive personality.

## Conclusions

The present study revealed the main predictors of employability, by undertaking a recent conceptual multifaceted model on employability that explored employability as an individual resource. Building on the model proposed by Lo Presti and Pluviano (2016), this study empirically tested the antecedents considered as potential influencing factors to achieving employability, namely training and work experiences, life events and circumstances, and personal dispositions. The research design focused on six variables that operationalised the three categories of predictors: educational level and the number of job roles for the *training and work experiences* factor, employability culture and family employability support for the *life events and circumstances* factor, and core self-evaluations and proactive personality for the *dispositions* factor. Assuming that employability might be determined by individual factors that the individual can control, such as education, as well as by contextual factors that are powered by the external environment (Berntson et al., 2006), the results brought evidence for the dominance of the predictors based on personal dispositions.

In line with the human capital approach (Becker, 1993), under the cluster *training and work experiences factor*s, the empirical findings showed that education was significantly and positively related to employability (H1 is supported). This is consistent with some of the previous findings (Berntson et al., 2006; Judhi et al., 2010) and might be consonant with the annotation that the relationship between education and employability is stronger, especially in periods of economic prosperity compared to periods of recession (Berntson et al., 2006). In terms of dominance analysis, educational level was dominant over all predictors except for personal dispositions, although its contribution in terms of explained variance was negligible. On the other hand, with respect to the work experience, we found that the number of previous job roles was not associated with employability (H2 not supported). This might be related to involuntary fluctuations and temporary contracts (Forrier & Sels, 2003b) and shows that unemployed persons hold a rather fragmented perspective on the flow of their work experiences.

With regard to the cluster of *life events and circumstances* as external factors that might be related to employability, the results showed that employability culture and family employability support were not associated with employability (H3 and H4 not supported). These findings stress the differences in the predictors of employability for individuals in employment and those unemployed. Whilst in the case of young students the relational support has a positive impact on the perceived employability (Cheung et al., 2018; Gunawan et al., 2020), these findings show that family support becomes less fruitful for the unemployed. This also shows that the mechanisms through which potential predicting factors behave might be different in the case of unemployed individuals compared to the employed population. As advocated by the existing employability models (Fugate et al., 2004; Lo Presti & Pluviano, 2016), these findings reveal employability being more strongly determined by internal predispositions than by external factors such as life circumstances.

In fact, the *dispositions* cluster revealed a positive and stronger impact on employability, being both core self-evaluations and proactive personality positively associated with employability (H5 and H6 supported). Our empirical findings showed that individuals with stronger proactive personality and more positive core self-evaluations exhibited higher employability, which presumably, was more strongly associated with heightened career success (Van der Heijden et al., 2009), psychological well-being (Gowan, 2012), quicker re-employment (Hennekam, 2015), and increased job satisfaction when getting re-employed (Gowan, 2012).

This study brings three main contributions. First, addressing the lack of studies that validate existing employability models, this research empirically tested the conceptual model on the antecedents of employability advanced by Lo Presti and Pluviano (2016). Secondly, considering the urge to explore employability skills from different perspectives (Arnedillo-Sánchez et al., 2018; Guilbert et al., 2016) and the identified differences in the way employability is experienced by different groups of individuals (e.g., employed vs. unemployed; Hennekam, 2015), this study shifted the focus from employed to unemployed persons and emphasised the need to examine employability as a personal resource for individuals in search of a job. Thirdly, the study addressed the need stressed in the literature to examine individual predictors that might influence employability (Wittekind et al., 2010). The findings shed light on a large variety of predictors and revealed the main antecedents of employability for unemployed persons. In particular, core self-evaluations and proactive personality were found to be significant and dominant predictors of employability. The evidence about core self-evaluations (Onyishi et al., 2015) and proactive personality (Gunawan et al., 2020) is consistent with previous studies, although we focused, for the first time, on a sample of job-seekers instead of students or employees. Future studies are needed to replicate such evidence of samples from other countries, also taking into account outcomes of employability for job-seekers (e.g., re-employment). Instead, mixed results were found in regards to educational level. In line with the literature that showed inconsistent evidence about the predictive role of educational level (Judhi et al., 2010; Nauta et al., 2009), educational level was positively associated with employability, although it showed a very low predictive power, compared to the other predictors when dominance analysis was concerned. Finally, no evidence was found in regards to the predictive power of work experience (consistently with the available mixed literature: Irwin et al., 2019; León & Morales, 2019), employability culture (contrary to available evidence: De Vos et al., 2011; Nauta et al., 2009), and family employability support.

As with any research, this study carried some limitations. The sample size was rather small, but comparable to other longitudinal studies that addressed employability longitudinally (McArdle et al., 2007; Wittekind et al., 2010). Nevertheless, research using larger samples across different contexts and nationalities is needed to validate these findings further. To assess employability, this study used a subjective measure of employability. Whilst the use of self-reports might imply inflation of relationships, by using a longitudinal research design at two different moments in time, the findings are prevented from suffering from the common method bias. Nevertheless, further studies could consider collecting information on employability from different sources, such as job centres counsellors. Also, in future research, control variables such as the length of unemployment and additional subsequent outcome variables could be included, such as the time needed to get re-employed, the salary level, or the job satisfaction with the new job following re-employment, as well as current job-search activities and efforts, career shocks. Finally, cross-lagged research design could allow assessing the reciprocal associations between variables overtime, at the same time controlling for autoregressive effects.

Given the complexity and breadth of the conceptual model, this study empirically tested only a limited set of measuring variables for the three predicting clusters. Further research should test other variables to operationalize the three clusters of antecedents encompassed in the employability conceptual model by Lo Presti and Pluviano (2016). Moreover, besides the independent value of each cluster of antecedents, there might be a synergy effect amongst the three categories of factors that impact individuals’ employability (Fugate et al., 2004; Lo Presti & Pluviano, 2016). Thus, we encourage future research to examine potential interactions between the components of the factors that determine employability.

### Theoretical implications

Building on the complexity of the conceptual model proposed by Lo Presti and Pluviano (2016), this study brings evidence for the differentiated impact of personal dispositions and situational resources on employability. Furthermore, the research sheds light on the distinct roles that the employability antecedents might play as a function to the individuals’ employment status and reveal potential changes in the impact of these antecedents along an individuals’ life. This study shows that individual dispositions, such as proactivity and core self-evaluations, as well as educational level, positively affect employability for unemployed persons as is the case of young students (Gunawan et al., 2020). By contrast, our results show that employability culture, family employability support, and previous work experience becomes insignificant for the unemployed. The findings reveal particular potential variations in the conceptual model on distinct categories of individuals and calls for further research to explore further aspects of the differentiated impact of employability antecedents.

### Practical implications

At an individual level, this study suggests that developing positive core self-evaluations (Judge et al., 2003) and a proactive approach when facing unemployment contributes to increased employability. Despite facing unemployment, the individuals with a better self-perceived ability to cope, to perform, and be successful, who believe they can control a broader array of factors in their lives, and show confidence and stability, will exhibit higher employability. Also, the unemployed persons who develop a stronger proactive attitude (Seibert et al., 1999), who allocate more resources for identifying opportunities and act on them, by aiming to change the circumstances instead of just passively adapting to the external conditions, show higher employability. Moreover, at the individual level, the findings bring evidence that education secures increased employability for unemployed persons.

On a social policy level, given that the individual attributes that build up employability were often addressed through single and fragmented policies and initiatives (Finn, 2000), this study brings informative findings to develop a more comprehensive approach for interventions aimed at increasing employability. Based on the empirical results, the policies addressed to unemployed persons should consider interventions targeted to the individual factors in addition to those focused on the structural conditions. The intervention programs need to design individual tailored programs, focused on assisting the unemployed in adopting a positive and proactive approach in their search for a job and incorporating coaching that covers the cognitive, emotional, and behavioural aspects of employability.

## Data Availability

Data are available from first author upon request.

## Code availability

Data have been processed through SPSS 21.

## References

[CR1] Arendt JN, Andersen HL, Saaby M (2020). The relationship between active labor market programs and employability of the long-term unemployed. Labour: Review of Labour Economics and Industrial Relations.

[CR2] Arnedillo-Sánchez I, De Aldama C, Tseloudi C (2018). rESSuME: Employability Skills Social Media SurvEy. International Journal of Manpower.

[CR3] Azen R, Budescu D (2003). The dominance analysis approach for comparing predictors in multiple regression. Psychological Methods.

[CR4] Beauregard, T. A. (2007). Family influences on the career life cycle. In M. Ozbilginand & A. Malach-Pines (Eds.), *Career Choice in Management and Entrepreneurship: A Research Companion* (pp. 101–126). Edward Elgar Press

[CR5] Becker, G. (1993). *Human capital: A theoretical and empirical analysis with special reference to education* (3rd Edn.). The University of Chicago Press.

[CR6] Berntson E, Marklund S (2007). The relationship between perceived employability and subsequent health. Work and Stress.

[CR7] Berntson E, Sverke M, Marklund S (2006). Predicting Perceived Employability: Human Capital or Labour Market Opportunities?. Economic and Industrial Democracy.

[CR8] Blustein DL, Chaves AP, Diemer MA, Gallagher LA, Marshall KG, Sirin S, Bhati KS (2002). Voices off the forgotten half: The role of social class in the school-to-work-transition. Journal of Counseling Psychology.

[CR9] Brown DJ, Cober RT, Kane K, Levy PE, Shalhoop J (2006). Proactive personality and the successful job search: A field investigation with college graduates. Journal of Applied Psychology.

[CR10] Cheng GHL, Chan DKS, Au WT (2020). Profiles of employability and their career and psychological implications among unemployed youth. Applied Research in Quality of Life.

[CR11] Cheung R, Jin Q, Cheung C (2018). Perceived employability of nonlocal Chinese university students in Hong Kong: The impact of acculturative and vocational variables. Journal of Career Assessment.

[CR12] Clarke M (2013). The organizational career: Not dead but in need of redefinition. International Journal of Human Resource Management.

[CR13] Crant JM (1995). The proactive personality scale and objective job performance among real estate agents. Journal of Applied Psychology.

[CR14] Crant JM (2000). Proactive behavior in organizations. Journal of Management.

[CR15] Darlington, R. B., & Hayes, A. F. (2016). *Regression analysis and linear models: Concepts, applications, and implementation*. Guilford Publications.

[CR16] DeFilippi RJ, Arthur MB (1994). The boundaryless career: A competency-based perspective. Journal of Orgaizational Behavior.

[CR17] De Vos A, De Hauw S, Van Der Heijden BIJM (2011). Competency development and career success: The mediating role of employability. Journal of Vocational Behaviour.

[CR18] Di Fabio A, Busoni L (2009). Proprietà psicometriche della versione italiana della Core Self-Evaluation Scale (CSES) con studenti di scuola secondaria” [Psychometric properties of the Italian version of the Core Self-Evaluation Scale (CSES) with high school students]. Counseling Giornale Italiano Di Ricerca e Applicazioni.

[CR19] Eby LT, Buch K (1994). The effect of job search method, sex, activity level, and emotional acceptance on new job characteristics: implications for counseling unemployed professionals. Journal of Employment Counseling.

[CR20] Eurostat (October, 2019) *Euro area unemployment*. Retrieved online: https://ec.europa.eu/.

[CR21] Fernández-Valera MM, Meseguer de Pedro M, De Cuyper N, García-Izquierdo M, Soler Sanchez MI (2020). Explaining job search behavior in unemployed youngsters beyond perceived employability: the role of psychological capital. Frontiers in Psychology.

[CR22] Ferry TR, Fouad NA, Smith PL (2000). The role of family context in social-cognitive model for career related choice behaviour: A math and science perspective. Journal of Vocational Behavior.

[CR23] Finn D (2000). From full employment to employability: A new deal for Britain’s unemployed?. International Journal of Manpower.

[CR24] Forrier A, De Cuyper N, Akkermans J (2018). The winner takes it all, the loser has to fall: Provoking the agency perspective in employability research. Human Resource Management Journal.

[CR25] Forrier A, Sels L (2003). The concept employability: A complex mosaic. International Journal of Human Resources Development and Management.

[CR26] Forrier A, Sels L (2003). Temporary employment and employability: Training opportunities and efforts of temporary and permanent employees in Belgium. Work Employment and Society.

[CR27] Fugate M, Kinicki AJ, Ashforth B (2004). Employability: A psycho-social construct, its dimensions, and applications. Journal of Vocational Behavior.

[CR28] Gazier, B. (Ed.). (1998). Employability-definitions and trends. In *Employability: Concepts and policies *(pp. 37–71). European Employment Observatory.

[CR29] Gowan MA (2012). Employability, well-being and job satisfaction following a job loss. Journal of Managerial Psychology.

[CR30] Guilbert L, Bernaud J, Gouvernet BE, Rossier J (2016). Employability: Review and research prospects. International Journal for Educational and Vocational Guidance.

[CR31] Gunawan W, Creed PA, Glendon AI (2020). Young adults’ perceived future employability: Antecedents and consequences. International Journal for Educational and Vocational Guidance.

[CR32] Hall DT (1996). Protean careers of the 21st century. Academy of Management Executive.

[CR33] Helyer R, Lee D (2014). The role of work experience in the future employability of higher education graduates. Higher Education Quarterly.

[CR34] Hennekam S (2015). Employability of older workers in the Netherlands: antecedents and consequences. International Journal of Manpower.

[CR35] Herbert IP, Rothwell AT, Glover JL, Lambert SA (2020). Graduate employability, employment prospects and work-readiness in the changing field of professional work. The International Journal of Management Education.

[CR36] Hobfoll SE (1989). Conservation of resources: A new attempt at conceptualizing stress. American Psychologist.

[CR37] Hodzic S, Ripoll P, Lira E, Zenasni F (2015). Can intervention in emotional competences increase employability prospects of unemployed adults?. Journal of Vocational Behavior.

[CR38] Hogan R, Chamorro-Premuzic T, Kaiser R (2013). Employability and career success: Bridging the gap between theory and reality. Industrial and Organizational Psychology.

[CR39] Irwin A, Nordmann E, Simms K (2019). Stakeholder perception of student employability: Does the duration, type and location of work experience matter?. Higher Education.

[CR40] Judge TA (2009). Core self-evaluations and work success. Current Directions in Psychological Science.

[CR41] Judge TA, Bono JE (2001). Relationship of core self-evaluations traits—self-esteem, generalized self-efficacy, locus of control, and emotional stability—with job satisfaction and job performance: A meta-analysis. Journal of Applied Psychology.

[CR42] Judge TA, Erez A, Bono JE, Thoresen CJ (2003). The Core Self-Evaluations Scale: Development of a measure. Personnel Psychology.

[CR43] Judge TA, VanVianen AE, DePater IE (2004). Emotional stability, core self-evaluations, and job outcomes: A review of the evidence and an agenda for future research. Human Performance.

[CR44] Judhi, N., Pa’Wan, F., Othman., N. A., & Moksin, H. (2010). Factors influencing internal and external employability of employees. *Business and Economics Journal*, 1–10. E-ISSN: 21516219

[CR45] Kirves K, Kinnunen U, De Cuyper N (2013). Contract type, perceived mobility and optimism as antecedents of perceived employability. Economic and Industrial Democracy.

[CR46] Koen J, Klehe UC, Van Vianen AE (2013). Employability among the long-term unemployed: A futile quest or worth the effort?. Journal of Vocational Behavior.

[CR47] León FR, Morales O (2019). The moderating role of tenure on the effects of job insecurity and employability on turnover intentions and absenteeism. Academia Revista Latino Americana De Administración.

[CR48] Lindstrom L, Doren B, Metheny J, Johnson P, Zane C (2007). Transition to employment: Role of the family in career development. Exceptional Children.

[CR49] Lo Presti A, Elia A (2020). Is the project managers’ road to success paved only with clear career paths? A dominance analysis of the additive contribution of career attitudes and employability factors. Project Management Journal.

[CR50] Lo Presti A, Ingusci E, Magrin ME, Manuti A, Scrima F (2019). Employability as a compass for career success: Development and initial validation of a new multidimensional measure. International Journal of Training and Development.

[CR51] Lo Presti A, Pluviano S (2016). Looking for a route in turbulent waters: Employability as a compass for career success. Organizational Psychology Review.

[CR52] McArdle S, Waters L, Briscoe JP, Hall DT (2007). Employability during unemployment: Adaptability, career identity and human and social capital. Journal of Vocational Behavior.

[CR53] McDaniel MA, Schmidt FL, Hunter JE (1988). Job experience correlates of job performance. Journal of Applied Psychology.

[CR54] McKee-Ryan FM, Song ZL, Wanberg CR, Kinicki AJ (2005). Psychological and physical well-being during unemployment: A meta-analytic study. Journal of Applied Psychology.

[CR55] McQuaid RW, Lindsay C (2005). The concept of employability. Urban Studies.

[CR56] Mincer J (1991). Education and unemployment. The National Bureau of Economic Research.

[CR57] Muehlboeck M, Steiber N, Kittel B (2020). Learning to keep the faith? Further education and perceived employability among young unemployed. Economic & Industrial Democracy.

[CR58] Nauta A, van Vianen A, Van Der Heijden B, Van Dam K, Willemsen M (2009). Understanding the factors that promote employability orientation: The impact of employabilityculture, career satisfaction, and role breadth self-efficacy. Journal of Occupational and Organizational Psychology.

[CR59] Ngo H, Liu H, Cheung F (2017). Perceived employability of Hong Kong employees: its antecedents, moderator and outcomes. Personnel Review.

[CR60] Norström F, Waenerlund A, Lindholm L, Nygren R, Sahlén K, Brydsten A (2019). Does unemployment contribute to poorer health-related quality of life among Swedish adults?. BMC Public Health.

[CR61] Onyishi IE, Enwereuzor IK, Ituma AN, Omenma JT (2015). The mediating role of perceived employability in the relationship between core self-evaluations and job search behaviour. Career Development International.

[CR62] Özer E, Hamarta E, Deniz ME (2016). Emotional intelligence, core-self evaluation, and life satisfaction. Psychology.

[CR63] Pavlin S, Svetlik I (2014). Employability of higher education graduates in Europe. International Journal of Manpower.

[CR64] Piccolo RF, Judge TA, Takahashi K, Watanabe N, Locke EA (2005). Core self-evaluations in Japan: Relative effects on job satisfaction, life satisfaction, and happiness. Journal of Organizational Behavior.

[CR65] Potgieter IL, Coetzee M, Masenge A (2012). Exploring employees’ personality attributes in relation to their employability attributes in the business management field. Journal of Psychology in Africa.

[CR66] Ryan NE, Solberg VS, Brown SD (1996). Family dysfunction, parental attachment, and career search self-efficacy among community college students. Journal of Counseling Psychology.

[CR67] Schlomer GL, Bauman S, Card NA (2010). Best practices for missing data management in counseling psychology. Journal of Counseling Psychology.

[CR68] Seibert SE, Crant JM, Kraimer ML (1999). Proactive personality and career success. Journal of Applied Psychology.

[CR69] Spurk D, Hirschi A, Dries N (2019). Antecedents and outcomes of objective versus subjective career success: Competing perspectives and future directions. Journal of Management.

[CR70] Trifiletti E, Capozza D, Pasin A, Falvo R (2009). A validation of the proactive personality scale. TPM -Testing, Psychometrics, Methodology in Applied Psychology.

[CR71] Vance RL, Coovert MD, MacCallum RC, Hedge JW (1989). Construct models of task performance. Journal of Applied Psychology.

[CR72] Van Dam K (2004). Antecedents and consequences of employability orientation. European Journal of Work and Organizational Psychology.

[CR73] Van der Heijden BIJM, DeLange AH, Demerouti E, Van Der Heijde CM (2009). Age effects on the employability–career success relationship. Journal of Vocational Behavior.

[CR74] Van Der Heijde CM, Van Der Heijden B (2006). A competence-based and multidimensional operationalization and measurement of employability. Human Resource Management.

[CR75] Vanhercke D, Kirves K, De Cuyper N, Verbruggen M, Forrier A, De Witte H (2015). Perceived employability and psychological functioning framed by gain and loss cycles. Career Development International.

[CR76] Virga D, De Witte H, Cifre E (2017). The role of perceived employability, core self-evaluations, and job resources on health and turnover intentions. The Journal of Psychology.

[CR77] Whiston SC, Keller BK (2004). The influences of the family of origin on career development: A review and analysis. The Counseling Psychologist.

[CR78] Williams N, Krasniqi B (2018). Coming out of conflict: How migrant entrepreneurs utilise human and social capital. Journal of International Entrepreneurship.

[CR79] Wittekind A, Raeder S, Grote G (2010). A longitudinal study of determinants of perceived employability. Journal of Organizational Behavior.

[CR80] World Medical Association (2001). Declaration of Helsinki: Ethical principles for medical research involving human subjects. Bulletin of the World Health Organization.

